# Supplementary material for: Complex‐centric proteome profiling by SEC‐SWATH‐MS
Source: Mol Syst Biol. 2019 Jan 14;15(1):e8438. doi: 10.15252/msb.20188438 (PMC6346213; doi:10.15252/msb.20188438)
Supplement: Supplementary file 8 — Dataset EV7 [file MSB-15-e8438-s008.zip › feature_plots_string/Q8IZP0.pdf]

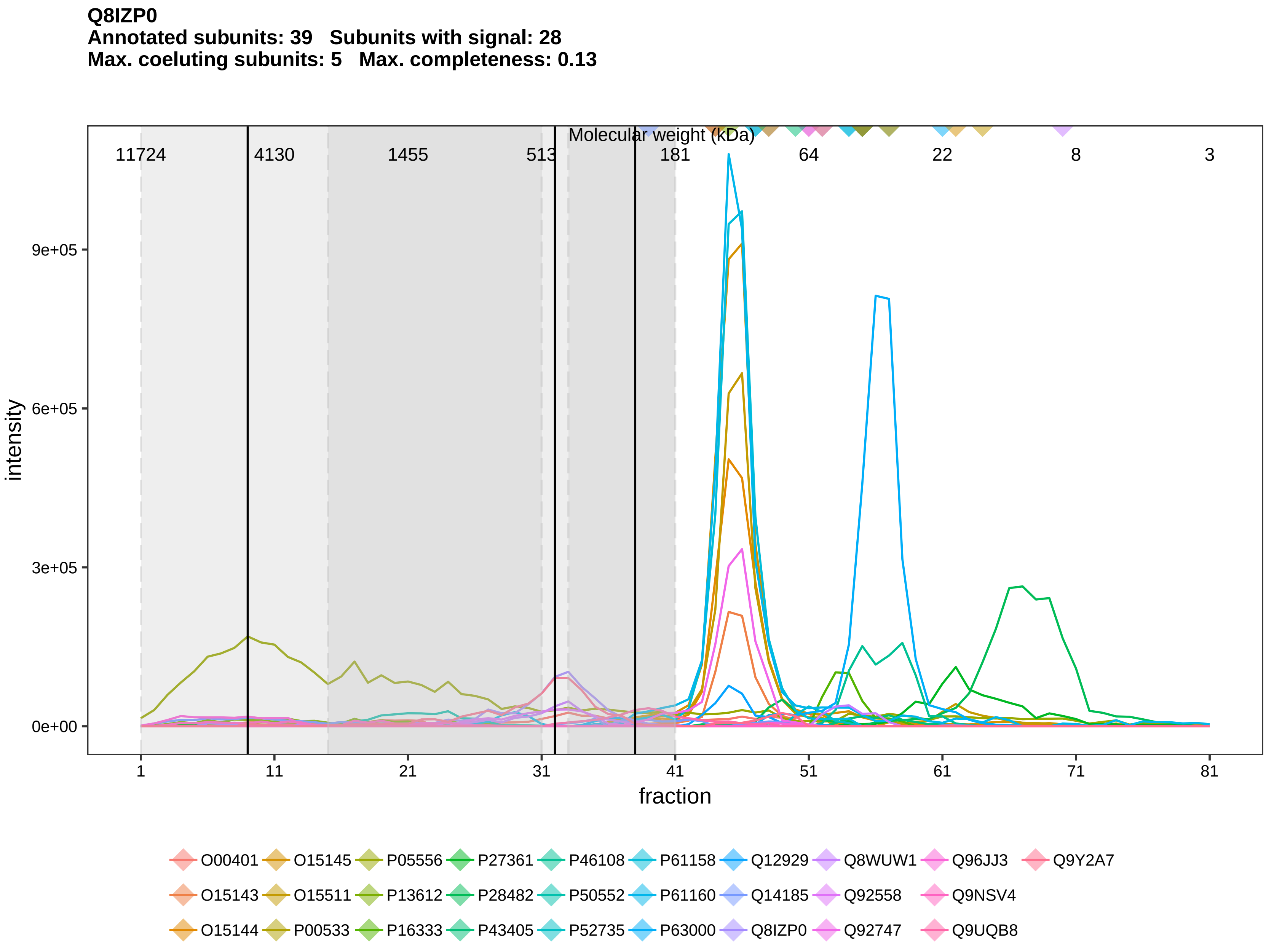

Q8IZP0
Annotated subunits: 39 Subunits with signal: 28
Max. coeluting subunits: 5 Max. completeness: 0.13
Molecular weight (kDa)
11724
4130
1455
513
181
64
22
8
3
intensity
9e+05
6e+05
3e+05
0e+00
fraction
1
11
21
31
41
51
61
71
81
O00401
O15143
O15144
O15145
O15511
P00533
P05556
P13612
P16333
P27361
P28482
P43405
P46108
P50552
P52735
P61158
P61160
P63000
Q12929
Q14185
Q8IZP0
Q8WUW1
Q92558
Q92747
Q96JJ3
Q9NSV4
Q9UQB8
Q9Y2A7
